# Isolation and Proteomic Analysis of Extracellular Vesicles from *Lactobacillus salivarius* SNK-6

**DOI:** 10.4014/jmb.2308.08017

**Published:** 2023-11-13

**Authors:** Jiwen Huang, Ayong Zhao, Daqian He, Xiao Wu, Huaxiang Yan, Lihui Zhu

**Affiliations:** 1 College of Animal Science and Technology, Zhejiang Agriculture and Forestry University, Hangzhou 311300, P.R. China; 2 Institute of Animal Husbandry and Veterinary Science, Shanghai Academy of Agricultural Sciences, Shanghai 201106, P.R. China; 3 Key Laboratory of Agricultural Genetics and Breeding, Biotechnology Research Institute, Shanghai Academy of Agricultural Sciences, Shanghai 201106, P.R. China

**Keywords:** Extracellular vesicles, *Lactobacillus salivarius*, protein profile

## Abstract

The proteins carried by the extracellular vesicles of *Lactobacillus salivarius* SNK-6 (LsEVs) were identified to provide a foundation for further explorations of the probiotic activities of *L. salivarius* SNK-6. LsEVs were isolated from the culture media of *L. salivarius* SNK-6 and morphological analysis was conducted by scanning electron microscopy. Subsequent transmission electron microscopy and nanoparticle tracking analysis were performed to assess the morphology and particle size of the LsEVs. In addition, the protein composition of LsEVs was analyzed using silver staining and protein mass spectrometry. Finally, internalization of the identified LsEVs was confirmed using a confocal microscope, and enzyme-linked immunosorbent assay was employed to analyze the levels of inflammatory cytokines in LPS-challenged RAW264.7 cells. The results revealed that the membrane-enclosed LsEVs were spherical, with diameters ranging from 100–250 nm. The LsEVs with diameters of 111–256 nm contained the greatest amount of cargo. In total, 320 proteins (10–38 kD) were identified in the LsEVs and included anti-inflammatory molecules, such as PrtP proteinase, co-chaperones, and elongation factor Tu, as well as some proteins involved in glycolysis/gluconeogenesis, such as fructose-1,6-bisphosphate aldolase. Enrichment analysis showed these proteins to be related to the terms “metabolic pathway,” “ribosome,” “glycolysis/gluconeogenesis,” “carbohydrate metabolism,” and “amino acid metabolism.” Furthermore, the LsEVs were internalized by host liver cells and can regulate inflammation. These findings confirm that LsEVs contain various functional proteins that play important roles in energy metabolism, signal transduction, and biosynthesis.

## Introduction

As a potential probiotic, *Lactobacillus salivarius* (*L. salivarius*) exhibits inhibitory effects on intestinal pathogens and regulates the equilibrium of the gut microbiome by producing various metabolites, antibacterial compounds, antioxidants, and organic acids [1, 2]. *L. salivarius* is also reported to alleviate diseases of the liver by reshaping the gut microbiota, regulating lipid metabolism and inflammation, and promoting lipid degradation [3-5]. However, the mechanisms employed by probiotics to alleviate fatty liver disease remain unclear and can differ among strains of the same species. A gram-positive strain previously isolated by us was identified as a new strain of *L. salivarius* and named SNK-6 [6]. In addition, *L. salivarius* SNK-6 was found to effectively inhibit deposition of fat droplets in the liver and regulate the peroxisome proliferator-activated receptor/sterol regulatory element-binding protein pathway, thereby preliminarily confirming that the strain could alleviate fatty liver disease in chickens, although the specific mechanisms remain unknown [6].

Included in the cargo of extracellular vesicles (EVs) released by the cell membrane of both gram-positive and -negative bacteria are proteins, lipids, polysaccharides, and nucleic acids that act as important mediators of communication with host cells and are implicated in the onset and progression of various diseases [7, 8]. However, most studies have focused on the EVs of gram-negative bacteria, mainly due to the presence of an outer membrane that more easily forms EVs, while research on EVs released by gram-positive bacteria has been limited [9]. In the current study, we isolated EVs from *L. salivarius* SNK-6 (LsEVs) and analyzed their protein components to enable further exploration of the function of *L. salivarius* SNK-6 and its EVs. Here, ultrafiltration and polymer precipitation methods were used to isolate EVs from *L. salivarius* SNK-6, which were then analyzed by transmission electron microscopy (TEM), nanoparticle tracking analysis (NTA), and silver staining of proteins. Additionally, the protein components of the LsEVs were identified by mass spectrometry. This study provided a theoretical basis for the use of *L. salivarius* SNK-6 as a probiotic in poultry production.

## Materials and Methods

### Isolation of LsEVs

**Fig. 1:**
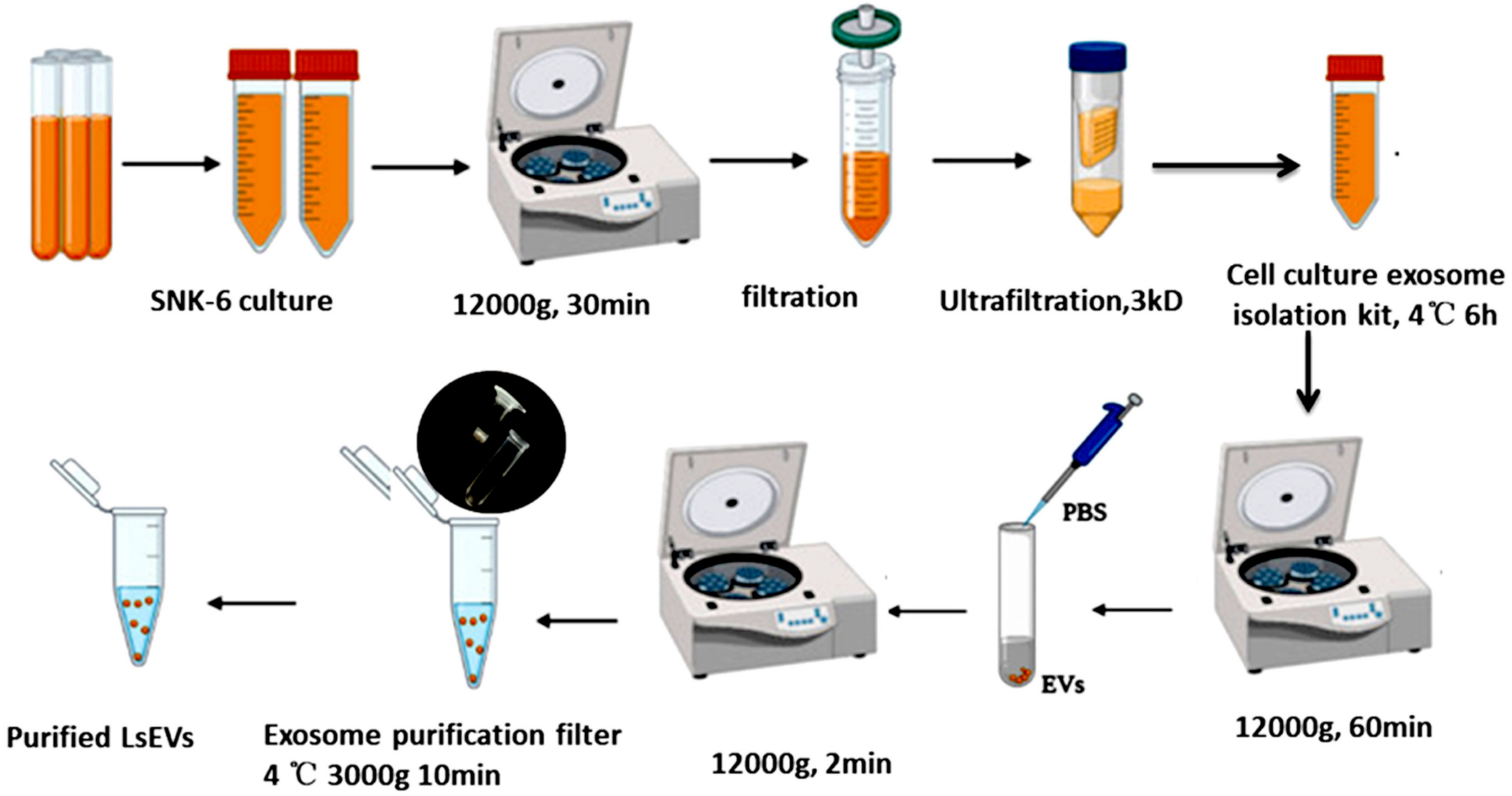
Schematic diagram of LsEV separation.

*L. salivarius* SNK-6 (GenBank, accession no. CP011403-CP011405) was maintained in our laboratory and routinely incubated in deMan–Rogosa–Sharpe medium at 41°C under anaerobic conditions [6]. LsEVs were isolated from the supernatant of *L. salivarius* SNK-6 cultured for 24 h by ultracentrifugation at 12,000 ×*g* for 20 min at 4°C, filtered with 0.45-μm bottle top filters (Corning Inc., USA), and then concentrated by centrifugation at 3,000 ×*g* for 30min at 4°C using a 3-kDa Ultrafiltration system (EMD Millipore Corp., Billerica, USA) (Fig. 1). The resulting pellets of LsEVs were collected using Invitrogen Total Exosome Isolation Reagent (from cell culture media) (Life Technologies, USA), and resuspended in diethylpyrocarbonate-treated water for NTA, TEM, and protein analysis. All experiments were conducted in accordance with the guidelines and regulations set forth by the Ethics and Animal Welfare Committee of the Shanghai Academy of Agricultural Sciences (approval no. SAASPZ0521022).

### Scanning Electron Microscopy (SEM) and TEM

The morphological characteristics of *L. salivarius* SNK-6 and LsEVs were assessed by SEM (SU8100; Hitachi High-Technologies Corp., Japan). Simply, *L. salivarius* SNK-6 cells were rinsed two times with phosphate-buffered saline, fixed with precooled osmic acid, dehydrated with alcohol, freeze-dried, and coated with gold nanoparticles using an ion sputtering instrument for SEM analysis (SU8100; Hitachi High-Technologies Corp.). In addition, the purified LsEVs were visualized by TEM (H-7600; Hitachi High-Technologies Corp.) as described previously [10]. Briefly, LsEVs were fixed in carboxylic acid buffer (pH 7.3). Then, fixed LsEVs (5 μl) were transferred to continuous carbon grids and negatively stained with 2% uranyl acetate. Samples were examined using an H-7600 transmission electron microscope (Hitachi High-Technology Corp.).

### Particle Size Analysis

The concentration and size of the LsEVs were measured by NTA (NanoSight NS300; Malvern Panalytical Ltd., UK) as described previously [11]. Briefly, samples were diluted at least 1:10 in PBS to obtain particles within a target measurement range of 10 to 100 particles per image on a Nanosight 300 (Malvern Panalytical Ltd.). Particle counting analysis was performed using Nanosight NTA 3.2 software (Malvern Panalytical Inc.) with a detection threshold of 5.

### Silver Staining

Aliquots (5 μl) of the prepared LsEVs were added to the wells of 4–20% polyacrylamide gels and the proteins were separated by electrophoresis. Then, the proteins were visualized by using a Silver Stain Kit (Sangon Biotech Co., Ltd., China).

### Cell Culture and EV Internalization

Human NCTC1469 hepatocytes were cultured in high-glucose Dulbecco’s modified Eagle’s medium (DMEM) supplemented with 10% horse serum and 1% penicillin/streptomycin at 37°C in 5% CO_2_. EVs (10 μg) were labeled with a working solution of PKH67 green fluorescent dye (2 μl) for 24 h. Subsequently, the nuclei of living cells were stained with 4’,6-diamidino-2-phenylindole (DAPI, Invitrogen). Finally, localization of the EVs was determined with a confocal microscope (Zeiss LSM 780, Germany).

### Proteomic Analysis of LsEVs

Triplicate samples of the LsEVs were mixed with protein lysis solution to dissolve the protein precipitate. The extracted proteins were digested overnight with trypsin (Promega Corporation, Madison, USA) at 37°C. The supernatant was collected and desalted using Strata-X columns (Phenomenex, Inc., USA). Subsequently, the samples were vacuum-dried and analyzed by liquid chromatography-mass spectrometry using an EASY-nLC 1200 UHPLC system (Thermo Fisher Scientific, USA) with an ACQUITY BEH C18 column (4.6 × 250 mm, 5 μm; Waters Corp., USA) coupled to a Q Exactive HF-X mass spectrometer (Thermo Fisher Scientific). The peptide mixtures were separated through an analytical C18 column (15 cm × 150 μm, 1.9 μm; Thermo Fisher Scientific). The raw data have been deposited in the ProteomeXchange Consortium database https://www.iprox.org (PXD044089).

### In Vitro Macrophage Assay

Murine macrophage RAW264.7 cells were cultured in the DMEM (Thermo Fisher Scientific) supplemented with 10% fetal bovine serum (FBS, Thermo Fisher Scientific) and 1% penicillin/streptomycin. The LPS-challenged RAW264.7 cells (5 × 10^5^ cells/ml) were pretreated with PBS or LsEVs (10 μg/ml) for 12 h and stimulated with PBS or LPS (1 μg/ml) for 12h. The cell culture media from the different treatments were collected to analyze the levels of cytokine IL-6 and TNF-α by enzyme-linked immunosorbent assay according to the manufacturer’s instructions (Sangon Biotech Co., Ltd., China). Four independent experiments were performed per treatment. Concentrations of the cytokines were calculated by referring to standard curves.

### Statistical Analysis

The resulting raw data were compared against the *L. salivarius* genome draft sequences retrieved from the NCBI and UniProt databases by Proteome Discoverer 2.4. The following parameters were used to filter peptides and proteins: maximum false discovery rate, 1%; peptide mass tolerance, 10 ppm; and fragment mass tolerance, 0.02 Da. Peptide spectrum matches (PSMs) with a confidence level greater than 99% are considered credible PSMs, and proteins containing at least one unique peptide are considered credible proteins. We only store trusted peptide and protein spectra and perform FDR validation to remove peptides and proteins with an FDR greater than 1%. Enrichment analysis of the identified proteins with Clusters of Orthologous Genes (COG) annotations in reference to the Kyoto Encyclopedia of Genes and Genomes (KEGG; https://www.genome.jp/kegg/) was performed using InterProScan software [12].

## Results

### *L. salivarius* SNK-6 Releases Nanosized EVs

**Fig. 2:**
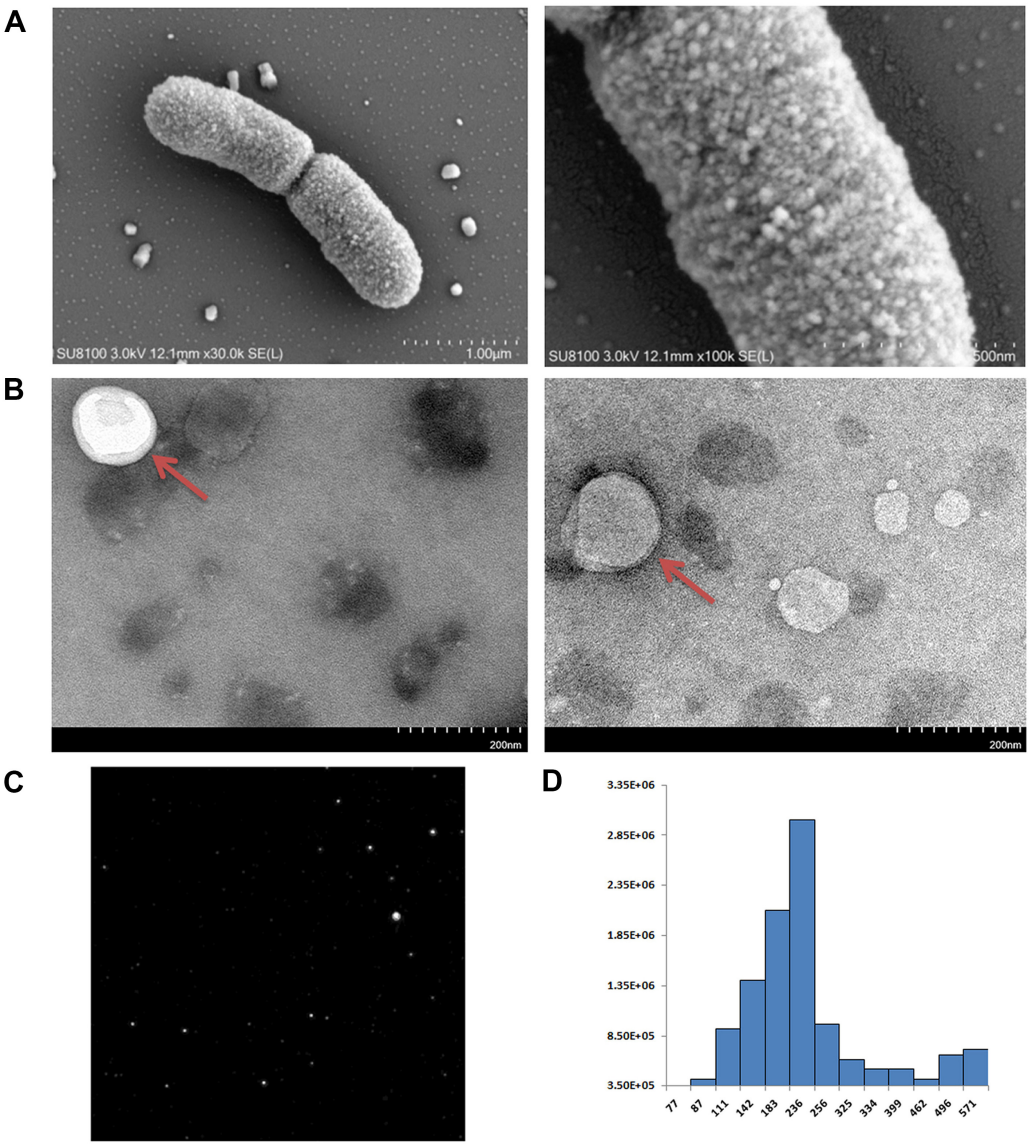
Characterization of EVs of *L. salivarius* SNK-6. (**A**) TEM images of purified EVs of *L. salivarius* SNK-6. (**B**) The size distribution of EVs of *L. salivarius* SNK-6. (**C**) Representative image of LsEVs characterized by NTA. (**D**) Size distribution of LsEVs. Abbreviations: EV, extracellular vesicle; TEM, transmission electron microscopy; NTA, nanoparticle tracking analysis.

The TEM images presented in Fig. 2A show that *L. salivarius* SNK-6 is a relatively short, rod-shaped bacterium arranged in pairs. Greater magnification revealed shedding of single and multiple EVs from the cell surface that contained various particles, as demonstrated by TEM and NTA (Fig. 2B and 2C). The purified LsEVs were spherical and enclosed in membranes. The majority of the EVs had diameters of 100–250 nm (Fig. 2D) with the largest measuring 236 nm. LsEVs ranging in size from 111 to 256 nm had the most cargo (Fig. 2D).

### Proteomic Analysis of LsEVs

**Fig. 3:**
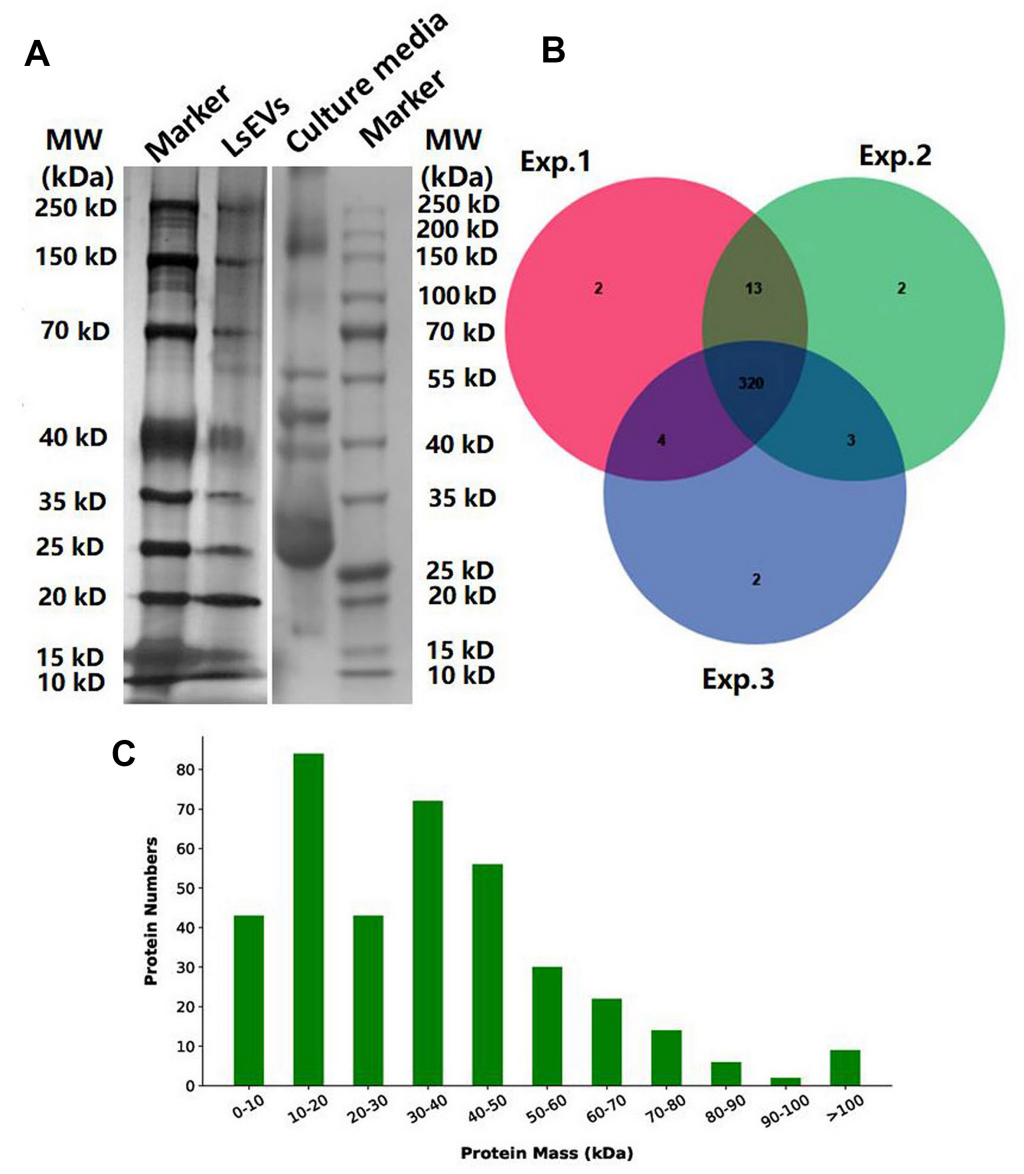
Biochemical and proteomic analyses of LsEVs. (**A**) Electrophoresis of purified proteins from the LsEVs. (**B**) Venn diagram of 320 overlapping proteins among triplicate samples. (**C**) Estimated mass of the proteins of LsEVs. Abbreviations: SDS-PAGE, sodium dodecyl sulfate polyacrylamide gel electrophoresis.

**Fig. 4:**
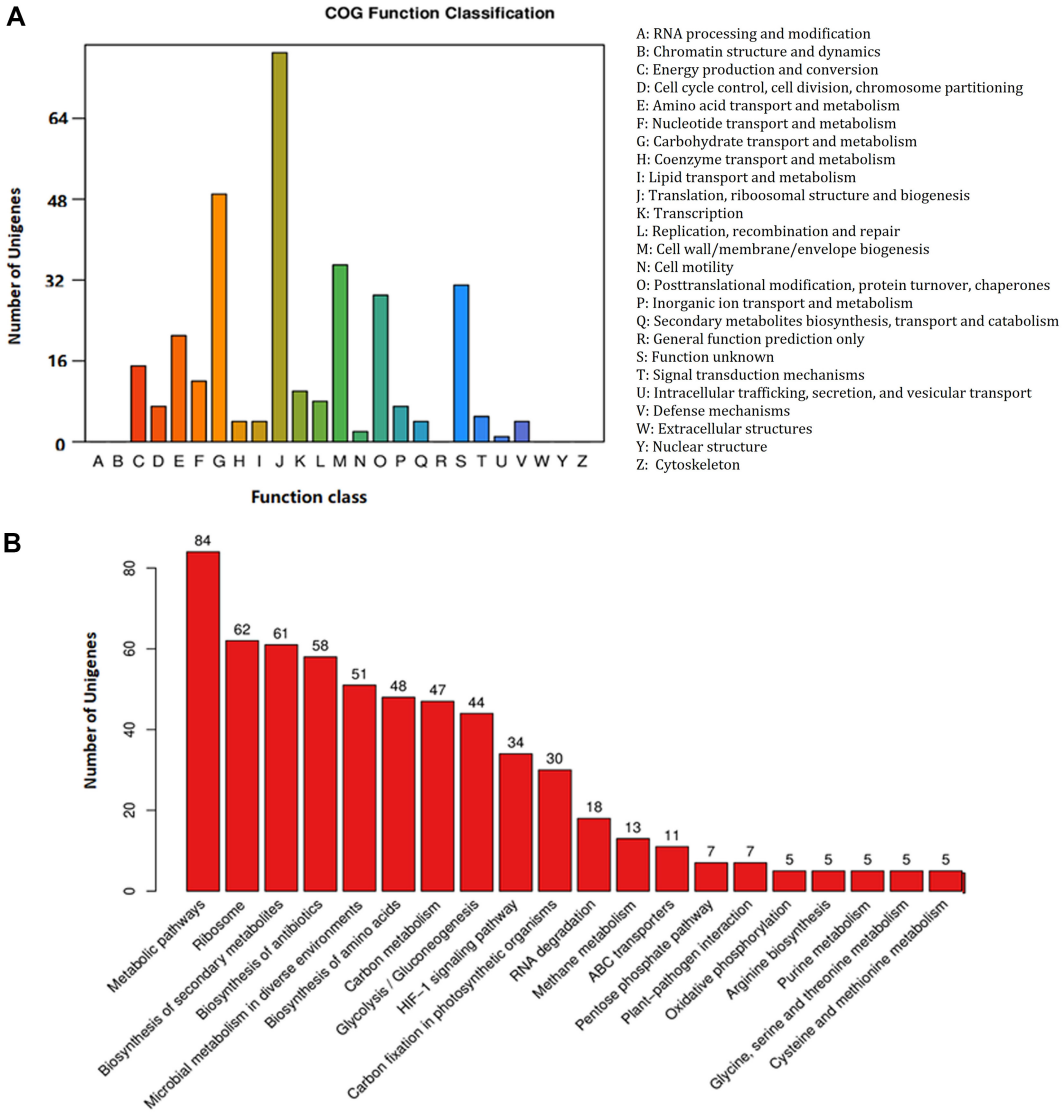
Functional analysis of the proteins of LsEVs. (**A**) COG terms associated with the identified proteins. (**B**) Top 20 KEGG pathways of the identified proteins.

The results of electrophoresis and silver staining clearly demonstrated the presence of proteins in the cargo of LsEVs (Fig. 3A). Proteomic analysis identified 320 overlapping proteins, mostly 10–50 kDa, among the triplicate samples of LsEVs (Fig. 3B and 3C). Most of the proteins were associated with the COG terms “carbohydrate transport and metabolism” and “cell wall/membrane/envelope biogenesis” (Fig. 4A). KEGG enrichment analysis showed that these proteins were associated with the terms “metabolic pathway,” “ribosome,” “glycolysis/gluconeogenesis,” “carbohydrate metabolism,” and “amino acid metabolism” (Fig. 4B). Notably, several of the shared proteins were classified as inflammation inhibitors, such as PrtP-encoded lactocepins, co-chaperones, serine protease inhibitor, and ubiquitin, while others were associated with glycolysis and gluconeogenesis, such as fructose-1,6-bisphosphate aldolase (FBA) and enolase (Table S1).

### LsEVs Were Internalized by Host Cells and Regulated Inflammation

**Fig. 5:**
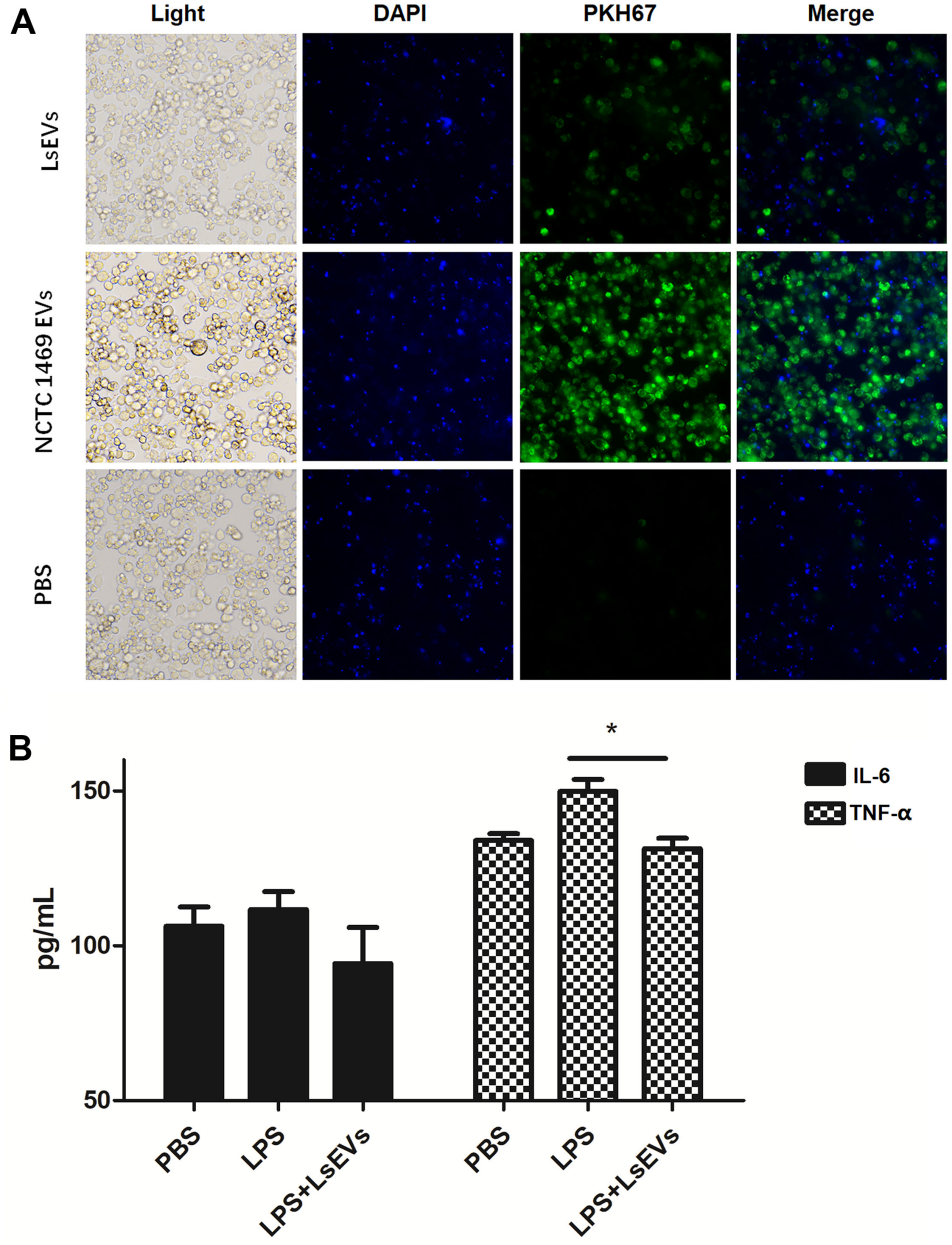
LsEVs could be internalized by host cells and regulate inflammation. (**A**) Internalization of LsEVs by host cells. Representative images of LsEVs incorporating PKH67 (green, 100 μM) and DAPI (blue, 10 μg/ml). (**B**) LsEVs regulate inflammation in LPS-activated RAW264.7 cells.

To confirm up-take by host cells, LsEVs were stained with the green fluorescent lipophilic compound PKH67, while the cell nuclei were labeled with DAPI. After co-incubation for 24 h, PKH67-labeled LsEVs were observed within the cytoplasm of NCTC1469 cells, demonstrating internalization by normal liver cells (Fig. 5A). Since several inflammation inhibitors were identified in LsEVs, we then added LsEVs in LPS-challenged RAW264.7 cells. We found that pretreatment with LsEVs markedly inhibited the elevated activity of TNF-α (*p* < 0.05) in LPS-activated RAW264.7 cells (Fig. 5B), although no significant difference in IL-6 level was observed between LPS-and LsEV-pretreated cells.

## Discussion

Most bacteria used as probiotics are members of the genus *Lactobacillus*. Although the roles of *L. salivarius* in disease prevention and treatment have been widely reported, the underlying mechanisms remain unclear. *L. salivarius* SNK-6 is reported to regulate metabolism of lipids in the liver, maintain balance of the intestinal microbiota, and enhance the intestinal immunity of laying hens [6, 13].

Bacterial extracellular vesicles (EVs) play a crucial role in facilitating both physiological and pathological functions. This is achieved through bacteria-bacteria and bacteria-host-cell interactions, as the EV cargo is transferred to receptor cells [14, 15]. Several technologies with particular advantages and disadvantages have been developed to separate EVs from cell culture media. These include ultracentrifugation, ultrafiltration, polymer precipitation, capture with immunomagnetic beads, and aptamer-based and microfluidic-based methods [16]. Ultracentrifugation is the most common method used for separation of EVs, but the procedure is time-consuming, requires expensive equipment, and recovery is variable [17]. Ultrafiltration, which does not require expensive equipment, is often used as an initial clean-up step to remove larger contaminants and has no effect on the biological activities of EVs. Polymer precipitation uses polymers to "hijack" water molecules, thereby decreasing the solubility of EVs and allowing sedimentation under low-speed centrifugal conditions [18]. Polymer precipitation is a simple, convenient, and fast method without the need for special equipment, as a regular centrifuge is sufficient, and the reagents are relatively inexpensive. In reference to a previously published method for separation of exosomes of *Schistosoma japonicum* [10], LsEVs were obtained in the present study by ultrafiltration combined with polymer precipitation. SEM and TEM images showed large numbers of single and multiple ellipsoidal EVs on the surfaces of *L. salivarius* SNK-6 cells with particle sizes of 50–200 nm, which was consistent with the morphology and size of *Lactobacillus* EVs reported in previous studies [19-21]. Moreover, NTA showed that the LsEVs had diameters of 100–250 nm, consistent with the observations by electron microscopy, further confirming the feasibility of the purification method used in this experiment.

A comprehensive understanding of the cargo carried by EVs is essential for clinical applications. As described in prior reports, proteomics analysis revealed various bioactive molecules as the cargo of EVs that influence the microbial population and host health [22, 23]. For example, EVs of *Pseudoalteromonas antarctica* NF3 contain degradative enzymes, synthetases, membrane-related proteins, and various proteins involved in metabolism and important biological processes in the bacterial cell, including growth, development, and signal transduction [22]. Rubio *et al*. [23] found that the EVs of *L casei* BL23 contain 103 proteins, including p40 and p75, which are related to probiotic effects, and LCABL_31160, an adhesion protein involved in bacterial colonization [23]. In another study, the EVs of *L. acidophilus* ATCC 53544 and *L. casei* ATCC 393 were analyzed, and a total of 395 and 201 proteins were identified, respectively. Among these proteins, 26 vesicular proteins were found in *L. acidophilus* ATCC 53544 EVs, while *L. casei* ATCC 393 EVs contained 43 vesicular proteins. Comparatively, 378 proteins, including 17 vesicular proteins, were found in the EVs of *L. reuteri* ATCC 23272. Of these, more than 80 proteins, including some with antibacterial activities, were detected in the EVs of the three conventional groups of *Lactobacillus* [24]. In the present study, 320 proteins from LsEVs were associated with the terms “metabolic pathway,” “ribosome,” and “glycolysis/gluconeogenesis.” In addition, these proteins were internalized by liver cells, hinting at the potential roles of LsEVs in host cells.

Notably, our results demonstrate that 320 proteins were identified in LsEVs, including co-chaperones, serine protease inhibitor, elongation factor Tu, adenosine triphosphate-binding cassette (ABC) transporter ATP-binding protein, glyceraldehyde-3-phosphate dehydrogenase (*GAPDH*), and ubiquitin, which were also identified in EVs in previous reports by Hu *et al*. [19], and lead us to further discussion. Among the enriched proteins identified in LsEVs, ABC transporter ATP-binding proteins are widely present in bacteria and are important for metabolite transport, drug resistance, and bacterial pathogenicity [25]. Among these, substrate-binding protein (SBP) is an important component of transport systems and functions in substrate recognition [26]. SBP-dependent ABC transporters differ among organisms and target a variety of substances, including metal ions, amino acids, peptides, and sugars, which are essential for the growth and survival of bacteria in different environments [27]. The LsEVs analyzed in this study were rich in proteins that bind to metal ions and amino acids as substrates of MetQ/NlpA family ABC transporter substrate-binding proteins, which are reportedly beneficial for colonization and survival of *L. salivarius* SNK-6 in vivo. *GAPDH* is a widely studied housekeeping protein that participates in glycolysis, cell metabolism, and maintaining cellular homeostasis [28]. Dysregulation of the glucose aerobic oxidation pathway has been linked to the occurrence of diabetes. Liu, *et al*. [29] identified 17 urinary exocrine proteins involved in glucose aerobic oxidation metabolism and found that *GAPDH* level is reduced in diabetes patients. Bai, *et al*. [30] reported that *Lactobacillus johnsonii* can enhance the integrity of the gut barrier in mice via interactions between *GAPDH* and tight junction proteins. PrtP is a cell envelope protease that is generally highly expressed in *Lactobacillus* and can inhibit the activities of bacteriocins, thereby exhibiting strong anti-inflammatory activities [31-33]. Elongation factor Tu is an immunoreactive protein highly expressed in most bacteria that mediates the adhesion of *Lactobacillus plantarum* HC-2 and might participate in gut homeostasis [34, 35]. Decreased level of FBA was correlated with poor prognosis of hepatocellular carcinoma [36]. Notably, quantitative proteomic analysis found that the enriched anti-inflammatory‐specific molecules in LsEVs included PrtP proteinase, co-chaperones, and elongation factor Tu, as well as proteins involved in glycolysis/gluconeogenesis, such as FBA and enolase [37, 38], which may be associated with the probiotic effects of *L. salivarius* SNK-6 in intestinal barrier repair and regulation of lipid metabolism in the liver [6, 13], although the underlying mechanisms have not yet been elucidated. Additionally, hypothetical protein B6U59_05510, of unknown function, was most enriched in LsEVs and needs further determination.

There were some limitations to this study that should be addressed. First, the effects of different separation methods on the protein cargo of LsEVs remain unclear. Second, to evaluate the potential benefits, safety, and efficacy of LsEVs, it is necessary to conduct animal studies and clinical trials. Third, the specific roles of the identified proteins on the interactions between bacteria and host cells must be further investigated. In the present study, we identified some proteins that may be related to immune regulation in LsEVs, such as 60 kDa chaperonin, elongation factor Tu, and ubiquitin. Since LsEVs were confirmed to inhibit the elevated activity of proinflammatory cytokine TNF-α in LPS-activated RAW264.7 cells, further studies will aim to investigate the precise molecules involved in the immunomodulatory effects of LsEVs. Finally, since we found that *L. salivarius* SNK-6 could effectively inhibit the deposition of fat droplets in the liver, and LsEVs secreted by *L. salivarius* SNK-6 could be internalized by host cells, the specific roles of LsEVs in regulating liver metabolism need further research.

In summary, LsEVs were successfully isolated from *L. salivarius* SNK-6. The cargo of LsEVs included more than 300 proteins that can be internalized by liver cells. The results of COG and KEGG enrichment analyses revealed that LsEVs may have significant roles in the interaction between bacteria and host cells. The proteins of LsEVs were mainly associated with carbohydrate metabolism, signal transduction, amino acid metabolism, and protein folding and degradation, indicating that LsEVs may regulate metabolism of glucose, amino acids, and lipids within the host cell. However, more research is required to fully understand the specific functions of these proteins.

## Supplemental Materials

Supplementary data for this paper are available on-line only at http://jmb.or.kr.
