# Supplementary material for: Isolation and Proteomic Analysis of Extracellular Vesicles from Lactobacillus salivarius SNK-6
Source: J Microbiol Biotechnol. 2023 Nov 13;34(1):224–31. doi: 10.4014/jmb.2308.08017 (PMC10840465; doi:10.4014/jmb.2308.08017)
Supplement: Supplementary file 1 [file jmb-34-1-224-supple.pdf]

J. Microbiol. Biotechnol.
https://doi.org/10.4014/jmb.2308.08017

Supplementary Table

Isolation and proteomic analysis of extracellular vesicles from *Lactobacillus salivarius* SNK-6

Jiwen Huang[1,2], Ayong Zhao[1*], Daqian He [2], Xiao Wu[3], Huaxiang Yan[2], and Lihui Zhu[2*]

[1]*College of Animal Science and Technology, Zhejiang Agriculture and Forestry University, Hangzhou 311300, China*
[2]*Institute of Animal Husbandry and Veterinary Science, Shanghai Academy of Agricultural Sciences, Shanghai 201106, China*
[3]*Key Laboratory of Agricultural Genetics and Breeding, Biotechnology Research Institute, Shanghai Academy of Agricultural Sciences, Shanghai 201106, China*

Lihui Zhu, Ph.D.

Email: zhulihui@saas.sh.cn.

Ayong Zhao, Ph.D.

Email: zay503@zafu.edu.cn.

Table S1. **List of protiens identified in LsEVs.**

NR_tophit_name	NR_Description	Coverage (%)	PSMs	Unique Peptides	AAs	MW (kDa)
OQQ86361.1	hypothetical protein B6U59_05510 [*Lactobacillus salivarius*]	37	625	32	827	86.8
HBU67548.1	type I glyceraldehyde-3-phosphate dehydrogenase [*Lactobacillus* sp.]	44	939	9	337	35.6
CAX65971.1	putative secreted protein [*Lactobacillus johnsonii* FI9785]	19	451	5	1423	151
KOY71545.1	Glyceraldehyde-3-phosphate dehydrogenase [*Lactobacillus kunkeei*]	4	78	1	337	36.3
EPH64255.1	glyceraldehyde-3-phosphate dehydrogenase, type I [*Enterococcus casseliflavus* 14-MB-W-14]	6	79	2	336	36.1
ADL50490.1	glyceraldehyde-3-phosphate dehydrogenase, type I [*Clostridium cellulovorans* 743B]	5	53	2	334	35.8
CAX67196.1	glyceraldehyde 3-phosphate dehydrogenase [*Lactobacillus johnsonii* FI9785]	12	294	2	338	36.3
AIR11321.1	Amino acid ABC transporter, substrate binding protein [*Lactobacillus salivarius*]	46	221	10	272	29.3
CDK35190.1	hypothetical protein LSCP400_09971 [*Lactobacillus salivarius* cp400]	54	90	3	46	5.3
CBL49691.1	Protein with YSRIK-signal peptide [*Lactobacillus crispatu*s ST1]	12	194	2	1898	201.8
HCI89943.1	50S ribosomal protein L30 [*Lactobacillus* sp.]	43	85	3	60	6.5
HBU68574.1	30S ribosomal protein S20 [*Lactobacillus* sp.]	24	3	1	84	9.1
AIR10972.1	Hypothetical protein LSJ_1310c [*Lactobacillus salivarius*]	14	42	1	175	18.4
ACL75228.1	protein of unknown function DUF214 [*Ruminiclostridium cellulolyticum* H10]	4	1	1	417	46.5
OQR18368.1	cell surface protein [*Lactobacillus salivarius*]	22	92	9	493	52
HBU67435.1	50S ribosomal protein L27 [*Lactobacillus* sp.]	35	105	2	93	9.9
HBU67575.1	co-chaperone GroES [*Lactobacillus* sp.]	53	61	4	94	10.3
KRM69818.1	endopeptidase [*Lactobacillus salivarius* DSM 20555 = ATCC 11741]	51	109	11	435	45.1
MYU50184.1	metal ABC transporter substrate-binding protein [*Lactobacillus salivarius*]	54	86	12	288	32.6
AIR09913.1	Cell surface protein [*Lactobacillus salivarius*]	40	113	5	209	23.4

SFG57506.1	glyceraldehyde 3-phosphate dehydrogenase [*Lactobacillus ruminis* DSM 20403 = NBRC 102161]	15	633	1	337	35.4
HBU67081.1	HU family DNA-binding protein [*Lactobacillus* sp.]	41	59	3	91	9.8
OQQ81763.1	hydrolase [*Lactobacillus salivarius*]	16	53	6	518	53
PEH09379.1	peptidoglycan-binding protein LysM [*Lactobacillus* sp. UMNPBX2]	30	40	4	189	20.1
ADJ79603.1	Sortase [*Lactobacillus salivarius* CECT 5713]	27	52	4	222	24.4
AIR10076.1	putative cell-wall-anchored protein (LPXTG motif) [*Lactobacillus salivarius*]	2	6	1	900	97
HBU67640.1	50S ribosomal protein L36 [*Lactobacillus* sp.]	59	14	1	37	4.3
OQR21718.1	hypothetical protein B6U40_01855 [*Lactobacillus salivarius*]	31	97	14	593	64.9
BAN47612.1	putative LysR family transcriptional regulator [*Pseudomonas resinovorans* NBRC 106553]	4	5	1	289	31.7
HBU68586.1	peptide deformylase [*Lactobacillus* sp.]	61	43	6	186	21.1
HBU68223.1	MetQ/NlpA family ABC transporter substrate-binding protein [*Lactobacillus* sp.]	45	80	10	271	29.8
KRM70725.1	50S ribosomal protein L12P [*Lactobacillus salivarius* DSM 20555 = ATCC 11741]	70	45	5	122	12.4
MYU48871.1	peptidylprolyl isomerase [*Lactobacillus salivarius*]	45	68	11	296	32.7
REE43218.1	glyceraldehyde 3-phosphate dehydrogenase [*Chryseobacterium* sp. 1335]	7	39	1	336	36.3
HBU68668.1	DNA/RNA non-specific endonuclease [*Lactobacillus* sp.]	37	42	6	276	30.4
ACA86158.1	response regulator with putative antiterminator output domain-like protein [*Shewanella woodyi* ATCC 51908]	6	1	1	181	20.3
AIR09834.1	LPXTG-motif cell wall anchor domain protein [*Lactobacillus salivarius*]	4	2	1	220	24.1
AHF16853.1	collagen-binding protein [*Niabella soli* DSM 19437]	1	4	1	1033	114.3
PZX38282.1	glyceraldehyde 3-phosphate dehydrogenase [*Nonlabens dokdonensis*]	4	40	1	334	36
AIR09812.1	Hypothetical protein LSJ_0041 [*Lactobacillus salivarius*]	50	34	6	131	14.5
ARW19073.1	50S ribosomal protein L31 type B [*Pediococcus pentosaceus*]	67	55	3	81	9.1
HCI89519.1	50S ribosomal protein L11 [*Lactobacillus* sp.]	30	25	2	141	14.8
HBU68449.1	50S ribosomal protein L32 [*Lactobacillus* sp.]	24	9	1	58	6.6
CDK36041.1	hypothetical protein LSCP400_18651 [*Lactobacillus salivarius* cp400]	64	28	5	118	13.3

MYU72651.1	acetyltransferase [*Lactobacillus salivarius*]	19	28	7	617	70.2
MYU49255.1	nucleoside 2-deoxyribosyltransferase [*Lactobacillus salivarius*]	48	34	4	155	17.3
AIR11542.1	Oligopeptide-binding protein oppA [*Lactobacillus salivarius*]	27	59	3	544	61.3
HBU67655.1	50S ribosomal protein L29 [*Lactobacillus* sp.]	41	28	1	64	7.6
AKH42982.1	General stress protein 39 [*Altererythrobacter atlanticus*]	4	14	1	292	31.3
MYZ22243.1	pantetheine-phosphate adenylyltransferase [*Lactobacillus salivarius*]	50	25	6	163	18.5
KRN21027.1	pgk protein [*Pediococcus claussenii*]	21	37	5	400	42.7
ADU21378.1	ubiquitin [*Ruminococcus albus* 7 = DSM 20455]	13	24	2	264	27.7
HBU67049.1	endolytic transglycosylase MltG [*Lactobacillus* sp.]	44	46	11	379	42.5
BAP17439.1	phosphoglycerate kinase [cyanobacterium endosymbiont of *Epithemia turgida* isolate EtSB Lake Yunoko]	3	47	1	400	42.9
BAQ45067.1	glucosamine--fructose-6-phosphateaminotransferase [*Methylobacterium aquaticum*]	3	8	1	608	64.9
HBU67350.1	thioredoxin [*Lactobacillus* sp.]	65	38	5	103	11.7
MYU70968.1	LTA synthase family protein [*Lactobacillus salivarius*]	17	27	7	689	78.4
HBU67313.1	rod shape-determining protein MreC [*Lactobacillus* sp.]	27	23	5	284	30.9
HBU67888.1	dTDP-4-dehydrorhamnose 3,5-epimerase [*Lactobacillus* sp.]	50	32	5	193	21.7
RFB37166.1	HU family DNA-binding protein [*Bacillus* sp. RC]	28	14	2	100	11
MYU70240.1	transcription elongation factor GreA [*Lactobacillus salivarius*]	54	16	5	156	17.4
OQR05787.1	peptide ABC transporter substrate-binding protein [*Lactobacillus salivarius*]	30	62	4	544	61.2
AIR11191.1	putative membrane spanning protein [*Lactobacillus salivarius*]	19	48	8	1008	105.2
HBW65924.1	molecular chaperone DnaK [*Chloroflexus aurantiacus*]	2	39	1	615	66.6
CDK34778.1	hypothetical protein LSCP400_05791 [*Lactobacillus salivarius* cp400]	20	27	7	641	70
ARW19154.1	50S ribosomal protein L7/L12 [*Pediococcus pentosaceus*]	33	23	3	121	12.4
HBU67652.1	50S ribosomal protein L24 [*Lactobacillus* sp.]	38	7	2	101	11
CDK36284.1	Glucose-6-phosphate isomerase (GPI) (Phosphoglucose isomerase) (PGI) (Phosphohexose isomerase) (PHI) [*Lactobacillus salivarius* cp400]	54	131	2	450	49.9

ARW20116.1	Phosphocarrier protein HPr [*Pediococcus pentosaceus*]	11	6	1	88	9.4
ACC73859.1	short-chain dehydrogenase/reductase SDR [*Paraburkholderia phymatum* STM815]	5	4	1	288	30.6
ALH81394.1	short-chain dehydrogenase [*Sphingopyxis macrogoltabida*]	4	10	1	311	32.7
PAY47606.1	hypothetical protein A8C55_00400 [*Lactobacillus salivarius*]	35	32	6	291	30.7
KRN20956.1	rplL protein [*Pediococcus claussenii*]	10	25	1	122	12.3
HBU68547.1	DNA-directed RNA polymerase subunit omega [*Lactobacillus* sp.]	91	15	5	65	7.2
MYZ22244.1	PDZ domain-containing protein [*Lactobacillus salivarius*]	25	23	7	344	37.8
ABD99953.1	Conserved hypothetical protein [*Lactobacillus salivarius* UCC118]	29	31	8	371	39.7
ADJ79655.1	Putative uncharacterized protein [*Lactobacillus salivarius* CECT 5713]	13	32	7	632	71.6
ATP36637.1	hypothetical protein CR249_10380 [*Lactobacillus salivarius*]	38	13	4	171	18.5
MYZ69142.1	PBP1A family penicillin-binding protein [*Lactobacillus salivarius*]	16	31	8	772	83.4
HBU68358.1	hypothetical protein [*Lactobacillus* sp.]	39	10	3	111	12.4
BAG13630.1	glyceraldehyde-3-phosphate dehydrogenase [uncultured Termite group 1 bacterium phylotype Rs-D17]	4	17	1	335	36
ALN87258.1	leupeptin-inactivating enzyme 1 [*Lysobacter capsici*]	2	1	1	670	70.4
CCC03205.1	50S ribosomal protein L31 [*Lactobacillus reuteri* ATCC 53608]	31	6	1	81	9.1
GEA97044.1	dTDP-4-dehydrorhamnose 3,5-epimerase [*Lactobacillus fermentum*]	20	20	1	193	21.7
ADI02348.1	histone family protein DNA-binding protein [*Syntrophothermus lipocalidu*s DSM 12680]	11	2	1	112	11.9
ADJ78380.1	ErfK/YbiS/YcfS/YnhG family protein [*Lactobacillus salivarius* CECT 5713]	9	19	4	446	49.6
ALX50411.1	glyceraldehyde-3-phosphate dehydrogenase [*Lentibacillus amyloliquefaciens*]	2	2	1	336	36.5
ATP36936.1	hypoxanthine phosphoribosyltransferase [*Lactobacillus salivarius*]	26	9	4	179	20.2
ATP36236.1	hypothetical protein CR249_08165 [*Lactobacillus salivarius*]	15	13	2	228	23.7
MYU93013.1	hypothetical protein [*Lactobacillus salivarius*]	21	21	3	165	19.7
OQR19768.1	succinate-semialdehyde dehydrogenase [*Lactobacillus salivarius*]	13	11	1	457	50.4
ADE30536.1	ribosomal protein S19 [*Streptococcus suis* GZ1]	20	12	2	123	14.1
CCK23570.1	30S ribosomal protein S10 [*Lactobacillus casei* W56]	23	10	2	111	12.7

MYU68476.1	pyridoxal phosphate-dependent aminotransferase [*Lactobacillus salivarius*]	38	36	10	393	43
MYZ00695.1	helix-turn-helix domain-containing protein [*Lactobacillus salivarius*]	3	3	1	308	33.5
ARU19562.1	ribosome-recycling factor [*Lactobacillus salivarius*]	36	19	4	187	21
AIR11481.1	PrtP proteinase [*Lactobacillus salivarius*]	20	49	13	1530	163.7
OWW46648.1	30S ribosomal protein S21 [*Enterococcus hirae* 81-15-F4]	15	3	1	65	7.7
KRN20165.1	pduJ protein [*Pediococcus claussenii*]	27	28	2	92	9.4
ANH02812.1	3-phosphoshikimate 1-carboxyvinyltransferase [*Shinella* sp. HZN7]	9	22	3	451	47
MYU68792.1	penicillin-binding protein 2 [*Lactobacillus salivarius*]	13	14	6	688	74.6
MYU69222.1	50S ribosomal protein L2 [*Lactobacillus salivarius*]	16	18	3	277	30
OQR05800.1	hypothetical protein B6U47_09440 [*Lactobacillus salivarius*]	8	7	1	130	15.3
MYU71209.1	polysaccharide deacetylase family protein [*Lactobacillus salivarius*]	30	22	5	266	30
ASC08922.1	10 kDa chaperonin [*Pediococcus pentosaceus*]	10	13	1	94	10.1
AGW12607.1	putative phosphopyruvate hydratase [*Desulfovibrio gigas* DSM 1382 = ATCC 19364]	3	3	1	441	47.1
MYU71210.1	hypothetical protein [*Lactobacillus salivarius*]	16	7	2	169	17.6
OUQ32543.1	rhodanese [*Lactobacillus salivarius*]	43	16	4	108	12.3
MYU68590.1	ASCH domain-containing protein [*Lactobacillus salivarius*]	77	21	6	149	17
AMP13460.1	ubiquitin-2 like Rad60 SUMO-like family protein [*Collimonas pratensis*]	24	18	1	76	8.5
HBU68216.1	dihydrolipoyl dehydrogenase [*Lactobacillus* sp.]	26	26	8	468	49.8
HBU67606.1	50S ribosomal protein L33 [*Lactobacillus* sp.]	22	5	1	50	5.8
HBU67255.1	4-oxalocrotonate tautomerase [*Lactobacillu*s sp.]	47	20	2	62	6.9
HBU68746.1	hypothetical protein [*Lactobacillus* sp.]	18	13	3	255	27.2
ATP37029.1	LytR family transcriptional regulator [*Lactobacillus salivarius*]	23	15	5	378	42.4
PAY61500.1	DNA polymerase III subunit beta [*Lactobacillus salivarius*]	34	17	7	379	41.9
ABE00249.1	Glutamine-binding protein [*Lactobacillus salivarius* UCC118]	16	10	3	276	30.1
CCK23558.1	50S ribosomal protein L24 [*Lactobacillus casei* W56]	9	2	1	106	11.7
ATP35559.1	transcriptional regulator [*Lactobacillus salivarius*]	24	22	1	324	36.8

HBU67199.1	hypothetical protein [*Lactobacillus* sp.]	39	17	2	66	7.6
CCH09267.1	hypothetical protein NH44784_053241 [*Achromobacter xylosoxidans* NH44784-1996]	4	1	1	224	24.5
HBU68164.1	fructose-1,6-bisphosphate aldolase, class II [*Lactobacillus* sp.]	18	11	3	288	31.1
ALJ23480.1	glyceraldehyde-3-phosphate dehydrogenase [*Lactobacillus gallinarum*]	13	7	1	338	36.5
OTF90190.1	transcriptional regulator [*Lactobacillus salivarius*]	25	23	1	324	36.8
AIR11213.1	N-acetylmuramoyl-L-alanine amidase [*Lactobacillus salivarius*]	6	12	4	938	104.9
SUE36443.1	2,3-bisphosphoglycerate-dependent phosphoglycerate mutase [*Rothia dentocariosa*]	4	2	1	257	29.2
ADJ78713.1	Glucose-6-phosphate isomerase (GPI) (Phosphoglucose isomerase) (PGI) (Phosphohexose isomerase) (PHI) [*Lactobacillus salivarius* CECT 5713]	52	131	1	450	49.9
WP_083776513.1	type I glyceraldehyde-3-phosphate dehydrogenase [*Desulfobacterium autotrophicum*]	4	2	1	339	37
AFD25487.1	Phosphoglycerate kinase [*Deinococcus gobiensis* I-0]	5	15	1	389	40.7
HBU67346.1	hypothetical protein [*Lactobacillus* sp.]	29	6	1	45	5.3
HCI89939.1	translation initiation factor IF-1 [*Lactobacillus* sp.]	58	13	3	72	8.3
ADJ79548.1	Hypothetical exported protein [*Lactobacillus salivarius* CECT 5713]	1	6	1	940	106.1
AIR09860.1	ATP-dependent clp protease ATP-binding subunit [*Lactobacillus salivarius*]	3	7	2	702	77.7
MYU49733.1	dihydroorotate dehydrogenase [*Lactobacillus salivarius*]	18	16	4	314	33.1
HBU67646.1	30S ribosomal protein S5 [*Lactobacillus* sp.]	39	22	4	166	17.3
ABD99884.1	GAF domain-containing proteins [*Lactobacillus salivarius* UCC118]	28	10	2	149	16.4
PAY33170.1	CamS family sex pheromone protein [*Lactobacillus salivarius*]	26	23	7	381	41.8
RMC52203.1	50S ribosomal protein L7/L12 [*Lactobacillus* sp. ESL0262]	29	6	1	121	12.5
BAG83358.1	30S ribosomal protein S10 [*Candidatus Azobacteroides pseudotrichonymphae genomovar*. CFP2]	12	3	1	103	11.7
AIR09968.1	Hypothetical protein, ErfK family [*Lactobacillus salivarius*]	12	3	2	246	28
ADU61522.1	enolase [*Pseudodesulfovibrio aespoeensis* Aspo-2]	3	9	1	429	46.1
ABQ66814.1	enolase [*Sphingomonas wittichii* RW1]	4	2	1	427	45.2
MSE04492.1	ABC transporter permease subunit [*Lactobacillus salivarius*]	20	19	7	501	54.5

AQR96836.1	enolase [*Clostridium saccharoperbutylacetonicum*]	4	6	1	430	47
BAN06622.1	50S ribosomal protein L27 [*Lactobacillus brevis* KB290]	10	1	1	115	12.4
BAU05229.1	hypothetical protein FIS3754_11230 [*Fischerella* sp. NIES-3754]	3	6	1	335	39.4
AAP95246.1	malate dehydrogenase [[*Haemophilus*] *ducreyi* 35000HP]	3	6	1	324	34.2
ADJ79142.1	Cell division protein [*Lactobacillus salivarius* CECT 5713]	16	7	3	284	32.3
HBU68249.1	peptide ABC transporter substrate-binding protein [*Lactobacillus* sp.]	14	17	3	330	35.1
ADJ78796.1	Chaperone protein dnaK (Heat shock protein 70) (Heat shock 70 kDa protein) (HSP70) [*Lactobacillus salivarius* CECT 5713]	6	8	2	615	66.5
AIR10693.1	Cytochrome d ubiquinol oxidase subunit I [*Lactobacillus salivarius*]	3	3	1	472	53
MYU69325.1	DUF4097 domain-containing protein [*Lactobacillus salivarius*]	12	11	3	321	35.1
ANX01974.1	type I glyceraldehyde-3-phosphate dehydrogenase [*Thermoclostridium stercorarium subsp. leptospartum* DSM 9219]	10	19	2	336	36.6
AGY81646.1	molecular chaperone DnaK [*Carnobacterium inhibens subsp. gilichinskyi*]	4	5	1	613	65.8
ARW19258.1	30S ribosomal protein S11 [*Pediococcus pentosaceus*]	22	11	2	129	13.7
MYZ22494.1	peptide ABC transporter substrate-binding protein [*Lactobacillus salivarius*]	25	75	1	537	59.7
KMT26918.1	glyceraldehyde-3-phosphate dehydrogenase Gap [*Melissococcus plutonius*]	5	3	1	336	36.5
HBU67630.1	50S ribosomal protein L13 [*Lactobacillus* sp.]	24	12	3	147	16.3
EIJ77824.1	glyceraldehyde-3-phosphate dehydrogenase [*Bacillus methanolicus* MGA3]	7	10	1	335	36.2
CDZ75213.1	DNA-binding protein HU [*Peptoniphilus* sp. ING2-D1G]	12	1	1	97	10.6
MYU69129.1	transporter substrate-binding domain-containing protein [*Lactobacillus salivarius*]	19	11	4	267	30
ABR47058.1	chaperonin Cpn10 [*Alkaliphilus metalliredigens* QYMF]	11	7	1	94	10.1
ADJ78417.1	D-alanyl-D-alanine serine-type carboxypeptidase [*Lactobacillus salivarius* CECT 5713]	19	22	2	385	42.3
ABD99699.1	Protein dltD precursor [*Lactobacillus salivarius* UCC118]	5	5	2	424	48.7
ASC08099.1	Fructose-bisphosphate aldolase [*Pediococcus pentosaceus*]	3	4	1	287	30.8
AIR10715.1	Penicillin binding protein 2B [*Lactobacillus salivarius*]	10	18	5	718	78.6
ADJ78503.1	Dipeptidase A [*Lactobacillus salivarius* CECT 5713]	14	11	4	472	53.9

ADJ79363.1	Putative secreted protein [*Lactobacillus salivarius* CECT 5713]	5	15	1	299	33.2
AKP67231.1	phosphoglyceromutase [*Lactobacillus ginsenosidimutans*]	5	4	1	229	25.9
AVL01357.1	50S ribosomal protein L33 [*Pediococcus pentosaceus*]	27	4	1	49	6
CBL21749.1	Rubrerythrin [*Blautia obeum* A2-162]	20	7	3	181	19.8
ADQ59318.1	arginine deiminase [*Lactobacillus amylovorus* GRL 1112]	7	7	2	423	48.1
ARU18596.1	oleate hydratase [*Lactobacillus salivarius*]	22	15	3	564	64.5
OQR21700.1	DUF4767 domain-containing protein [*Lactobacillus salivarius*]	6	6	2	364	39.9
HBU67644.1	50S ribosomal protein L15 [*Lactobacillus* sp.]	24	7	3	144	15.6
ARW18951.1	Stress response regulator gls24 like protein [*Pediococcus pentosaceus*]	7	11	1	136	15.2
MRG68597.1	urease subunit alpha [*Lactobacillus reuteri*]	2	7	1	573	61.8
HBU67437.1	50S ribosomal protein L21 [*Lactobacillus* sp.]	29	6	2	102	11.3
OQR19259.1	hypothetical protein B6U40_07655 [*Lactobacillus salivarius*]	7	12	2	924	97.4
ABJ58335.1	translation elongation factor 1A (EF-1A/EF-Tu) [*Lactobacillus delbrueckii* subsp. *bulgaricus* ATCC BAA-365]	12	16	1	396	43.3
AIR11379.1	Oligopeptide-binding protein oppA [*Lactobacillus salivarius*]	28	82	1	537	59.7
AKG45830.1	dna modification methylase [*Streptomyces xiamenensis*]	3	6	1	354	38.6
CDK34447.1	putative Zn-dependent protease [*Lactobacillus salivarius* cp400]	21	9	3	220	24.9
OQR20014.1	cellulose synthase [*Lactobacillus salivarius*]	6	9	3	690	78.2
RYQ48527.1	glyceraldehyde-3-phosphate dehydrogenase [*Bifidobacterium pseudolongum* subsp. *globosum*]	3	5	1	352	38
ALF58865.1	serine protease inhibitor [*Psychrobacter urativorans*]	2	1	1	451	50.2
EIA33423.1	hypothetical protein SMXD51_00289 [*Lactobacillus salivarius* SMXD51]	51	9	3	73	8.4
ADJ78621.1	Hypothetical secreted protein [*Lactobacillus salivarius* CECT 5713]	3	5	1	345	38.6
HBU67647.1	50S ribosomal protein L18 [*Lactobacillus* sp.]	19	3	2	118	12.8
CCG26795.1	SSU ribosomal protein S16p [*Streptococcus pyogenes* NS88.2]	22	1	1	90	10.2
MYU69250.1	RND transporter MFP subunit [*Lactobacillus salivarius*]	18	17	4	357	38.8

ADN13451.1	Phosphoglycerate kinase [*Gloeothece verrucosa* PCC 7822]	4	3	1	401	42.4
KRN20259.1	rpsI protein [*Pediococcus claussenii*]	14	3	1	130	14.4
HBU68438.1	50S ribosomal protein L35 [*Lactobacillus* sp.]	15	5	1	66	7.7
KRK90704.1	molecular chaperone GroEL [*Lactobacillus koreensis* JCM 16448]	7	13	1	541	57.1
OSH11851.1	LSU ribosomal protein L31P [*Enterococcus faecalis*]	14	1	1	99	11.2
AER17706.1	Ribosomal protein L31 [*Streptococcus suis* D9]	10	2	1	134	15.7
STX16981.1	co-chaperonin GroES [*Lactobacillus acidophilus*]	17	4	1	94	10.2
ADJ78387.1	Putative uncharacterized protein [*Lactobacillus salivarius* CECT 5713]	10	7	2	266	30.6
MYU71694.1	D-alanyl-D-alanine carboxypeptidase [*Lactobacillus salivarius*]	17	15	4	384	42.3
KRN20953.1	rplK protein [*Pediococcus claussenii*]	16	21	1	141	14.7
CDK34828.1	SSU ribosomal protein S16p [*Lactobacillus salivarius* cp400]	18	8	1	91	10.5
AIR09820.1	YycH protein [*Lactobacillus salivarius*]	9	7	3	445	50.6
AZK59593.1	Selenocysteine-specific translation elongation factor [*Candidatus Desulforudis audaxviator*]	3	1	1	635	70.2
PAY25265.1	cell division protein DIVIC [*Lactobacillus salivarius*]	11	4	1	131	15.6
AHH22071.1	putative ROK family transcriptional regulator [*Nocardia nova* SH22a]	2	1	1	399	41.7
KRM17858.1	30S ribosomal protein S12 [*Lactobacillus hayakitensis* DSM 18933 = JCM 14209]	18	8	2	137	15.1
HBU68744.1	50S ribosomal protein L9 [*Lactobacillus* sp.]	8	4	1	149	17.1
OQQ76630.1	DUF4811 domain-containing protein [*Lactobacillus salivarius*]	14	10	1	233	26.4
AJO74352.1	molecular chaperone DnaK [*Lactobacillus plantarum*]	4	10	1	622	66.7
AHB70753.1	hypothetical protein P262_03354 [*Cronobacter malonaticus*]	6	7	1	334	36
QHM66609.1	Enolase 2 [*Pediococcus pentosaceus*]	10	3	2	440	47.6
ABD01114.1	antioxidant, AhpC/Tsa family [*Synechococcus* sp. JA-2-3B'a(2-13)]	5	4	1	216	24.3
HBU68480.1	DUF896 family protein [*Lactobacillus* sp.]	31	8	3	84	10
AMA55925.1	DNA mismatch repair protein MutL [*Bradyrhizobium* sp. CCGE-LA001]	2	1	1	604	65.3
MYU68547.1	pyridoxal phosphate-dependent aminotransferase [*Lactobacillus salivarius*]	15	10	4	387	43.2

HBU68374.1	N-acetylneuraminate lyase [*Lactobacillus* sp.]	10	10	2	290	32.5
PNH19639.1	molecular chaperone GroEL [*Mageeibacillus indolicus*]	2	1	1	543	57.7
EEJ74401.1	hypothetical protein HMPREF0545_0605 [*Lactobacillus salivarius* DSM 20555 = ATCC 11741]	19	3	1	86	10.1
AIR10675.1	putative membrane spanning protein [*Lactobacillus salivarius*]	3	5	1	325	36.8
ARW19794.1	Cell cycle protein GpsB [*Pediococcus pentosaceus*]	14	7	1	113	12.8
AIR10111.1	hypothetical protein LSJ_0371c [*Lactobacillus salivarius*]	12	1	1	95	10.9
KRN19483.1	rpmA protein [*Pediococcus claussenii*]	16	1	1	95	10.4
ABR50723.1	Desulfoferrodoxin, ferrous iron-binding region [*Alkaliphilus metalliredigens* QYMF]	8	3	1	124	13.8
SMQ83724.1	phosphoglycerate kinase [*Synechococcus* sp. 7002]	3	2	1	398	41.5
ABJ64742.1	LSU ribosomal protein L36P [*Lactobacillus brevis* ATCC 367]	59	10	1	39	4.5
ADJ79132.1	Peptidoglycan binding protein, LysM domain protein [*Lactobacillus salivarius* CECT 5713]	16	4	2	217	23.3
AIR11192.1	hypothetical protein LSJ_1542c [*Lactobacillus salivarius*]	11	5	2	315	34.6
AGA25579.1	urease, alpha subunit [*Singulisphaera acidiphila* DSM 18658]	3	3	1	573	61.1
MYU69416.1	NAD-dependent succinate-semialdehyde dehydrogenase [*Lactobacillus salivarius*]	17	12	1	457	50.3
MYU69473.1	LTA synthase family protein [*Lactobacillus salivarius*]	6	8	3	727	82.5
PAY63793.1	hypothetical protein A8C45_04545 [*Lactobacillus salivarius*]	13	7	3	333	38.1
HBU68486.1	50S ribosomal protein L33 [*Lactobacillus* sp.]	39	3	1	49	6
ALV22258.1	SSU ribosomal protein S8p [*Carnobacterium* sp. CP1]	8	5	1	132	14.7
ATY83691.1	elongation factor Tu [*Kyrpidia spormannii*]	15	24	1	396	43.8
ATP37013.1	ABC transporter permease [*Lactobacillus salivarius*]	2	2	1	406	43.9
HBU68231.1	nitroreductase family protein [*Lactobacillus* sp.]	19	6	3	214	24.1
QEX45841.1	type I glyceraldehyde-3-phosphate dehydrogenase [*Mycoplasma gallisepticum*]	4	1	1	342	37.3
BAM86442.1	ATP synthase, F1 beta subunit [*Bradyrhizobium oligotrophicum* S58]	3	2	1	480	51.2
AFM42108.1	hypothetical protein Desaci_3207 [*Desulfosporosinus acidiphilus* SJ4]	17	5	1	86	8.6

AIR11473.1	Hypothetical protein LSJ_2056 [*Lactobacillus salivarius*]	17	5	2	187	21.9
HBU67155.1	phosphocarrier protein HPr [*Lactobacillu*s sp.]	14	7	1	88	9.3
OQR17175.1	glutamine ABC transporter substrate-binding protein [*Lactobacillus salivarius*]	3	6	1	485	53.3
HCD08622.1	30S ribosomal protein S21 [*Lactobacillus* sp.]	17	2	1	60	7.3
HCI90508.1	cold-shock protein [*Lactobacillus* sp.]	29	2	1	66	7.4
HBU67545.1	enolase [*Lactobacillus* sp.]	11	7	2	441	48
HBU68286.1	pyruvate kinase [*Lactobacillus* sp.]	5	3	2	586	62.8
AKZ56534.1	phosphoglyceromutase 1 [*Streptomyces ambofaciens* ATCC 23877]	4	1	1	253	28.2
CDK35943.1	hypothetical protein, lipoprotein [*Lactobacillus salivarius* cp400]	4	2	1	273	31.6
EGJ49681.1	hypothetical protein Desaf_1342 [*Desulfocurvibacter africanus subsp. africanus str. Walvis Bay*]	9	4	1	135	15.1
OLF84246.1	O-succinylhomoserine sulfhydrylase [*Marinobacter sp*. C18]	4	1	1	413	44.7
BAR77739.1	DNA-binding protein HU [*Bacillus anthracis*]	22	9	1	117	12.9
HCS31405.1	stage V sporulation protein S [*Eubacterium* sp.]	13	2	1	86	8.9
RNM49951.1	50S ribosomal protein L11 [*Staphylococcus aureus*]	10	2	1	140	14.8
AIR10709.1	Cell division protein [*Lactobacillus salivarius*]	3	2	1	419	44.4
AFS71373.1	Flagellin domain protein [*Exiguobacterium antarcticum* B7]	4	2	1	297	32.3
AFQ45764.1	flagellin/flagellar hook associated protein [*Desulfosporosinus meridiei* DSM 13257]	2	1	1	823	83.7
ATP37390.1	spermidine/putrescine ABC transporter substrate-binding protein [*Lactobacillus salivarius*]	9	6	2	361	41.5
AOO72993.1	GMP synthase [*Lactobacillus salivarius*]	5	3	1	221	24.7
AXR43957.1	50S ribosomal protein L10 [*Pediococcus pentosaceus*]	16	3	2	168	17.9
CEF19133.1	Phage major capsid protein [*Staphylococcus xylosus*]	3	1	1	443	49.5
PAY50932.1	hypothetical protein A8C37_09580 [*Lactobacillus salivarius*]	10	7	2	401	42.9
KRN20236.1	hypothetical protein IV79_GL000903 [*Pediococcus claussenii*]	41	15	1	64	7.6
AKJ70732.1	succinate-semialdehyde dehydrogenase (NADP(+)) [*Pandoraea thiooxydans*]	2	5	1	499	53.1
MYU72985.1	extracellular solute-binding protein [*Lactobacillus salivarius*]	9	10	3	431	49.4

HBU68579.1	elongation factor Tu [*Lactobacillus* sp.]	17	21	1	395	43.2
KGO16262.1	adenylate kinase [*Oenococcus oeni* X2L]	6	1	1	188	20.7
AXR42881.1	nitroreductase [*Pediococcus pentosaceus*]	5	2	1	201	22.5
TXJ80713.1	oleate hydratase [*Lactobacillus salivarius*]	25	15	1	384	44.2
BAB81005.1	enolase [*Clostridium perfringens* str. 13]	4	3	1	431	46.9
AGK98502.1	S-adenosylmethionine synthetase [*Clostridium pasteurianum* BC1]	4	2	1	402	43.8
HBU67518.1	tRNA (uridine(34)/cytosine(34)/5-carboxymethylaminomethyluridine(34)-2'-O)-methyltransferase TrmL [*Lactobacillus* sp.]	13	2	1	170	19.5
CCJ19128.1	S-adenosylmethionine synthase [*Staphylococcus aureus subsp. aureus* ST228]	3	3	1	432	47.8
BAP56480.1	proteinase inhibitor I4, serpin [*Thioploca ingrica*]	2	4	1	427	47.8
HBU68120.1	30S ribosomal protein S6 [*Lactobacillus* sp.]	10	7	1	96	11
AFI83612.1	phosphogluconate dehydrogenase (NADP(+)-dependent, decarboxylating) [*Methylophaga nitratireducenticrescens*]	8	7	2	488	53.3
RWA97799.1	50S ribosomal protein L7/L12 [*Mesorhizobium* sp.]	11	6	1	125	12.8
STX16946.1	LSU ribosomal protein L7/L12 (P1/P2) [*Lactobacillus acidophilus*]	17	5	1	120	12.5
MYU71070.1	ATP-binding cassette domain-containing protein [*Lactobacillus salivarius*]	2	3	1	661	72.7
OQR21058.1	hypothetical protein B6U40_03550 [*Lactobacillus salivarius*]	8	2	1	167	19.4
HBU67315.1	cold-shock protein [*Lactobacillus* sp.]	13	3	1	69	7.7
AEW72978.1	Outer membrane protein A [*Enterobacter ludwigii*]	3	5	1	380	41
OQR18938.1	peptide ABC transporter substrate-binding protein [*Lactobacillus salivarius*]	22	63	1	537	59.7
AYW51091.1	30S ribosomal protein S16 [*Tetragenococcus halophilus*]	13	3	1	91	10.4
BAU43660.1	Transketolase [*Leptolyngbya* sp. O-77]	2	1	1	708	75.9
ACL75593.1	Carbohydrate binding family 6 [*Ruminiclostridium cellulolyticum* H10]	2	1	1	604	64.9
AEP13522.1	Short-chain dehydrogenase of various substrate specificities [*Chloracidobacterium thermophilum* B]	4	2	1	278	29.8

OSP17005.1	type I glyceraldehyde-3-phosphate dehydrogenase [*Staphylococcus agnetis*]	4	1	1	335	36.3
ADJ79001.1	Signal peptidase I [*Lactobacillus salivarius* CECT 5713]	5	1	1	218	24.6
ASC07827.1	Acid shock protein [*Pediococcus pentosaceus*]	9	3	1	140	16
MYU69308.1	sugar ABC transporter substrate-binding protein [*Lactobacillus salivarius*]	3	4	1	330	37.1
CDZ75024.1	30S ribosomal protein S2 [*Peptoniphilus* sp. ING2-D1G]	5	2	1	275	31.4
HBU67639.1	30S ribosomal protein S13 [*Lactobacillus* sp.]	18	2	2	121	13.5
ESS01305.1	50S ribosomal protein L16 [*Lactobacillus fermentum* NB-22]	10	3	1	144	16.1
AIR11523.1	Choloylglycine hydrolase [*Lactobacillus salivarius*]	12	3	2	324	36.5
ADJ78415.1	Hypothetical membrane spanning protein [*Lactobacillus salivarius* CECT 5713]	2	4	1	598	66.9
HBU68534.1	ATP synthase subunit alpha [*Lactobacillus* sp.]	2	2	1	503	54.7
AIY04421.1	molecular chaperone GroEL [*Planococcus* sp. PAMC 21323]	7	10	1	543	57.4
AFS00147.1	30S ribosomal protein S2 [*Lactobacillus buchneri* CD034]	3	2	1	288	32.4
BAP86223.1	molecular chaperone GroEL [*Lactobacillus hokkaidonensis* JCM 18461]	12	14	1	542	57.1
ADH85803.1	Ketol-acid reductoisomerase [*Desulfurivibrio alkaliphilus* AHT 2]	4	5	1	490	53.8
MYU69455.1	dipeptidase PepV [*Lactobacillus salivarius*]	5	4	2	469	51.5
CDF74442.1	10 kDa chaperonin [*Lactobacillus acidophilus* DSM 20242]	13	3	1	94	10.3
PAY53356.1	hypothetical protein A8C37_05445 [*Lactobacillus salivarius*]	8	3	1	209	23.7
MYU49306.1	hypothetical protein [*Lactobacillus salivarius*]	5	2	1	265	30.7
CUP00114.1	ribosome recycling factor [*Clostridium baratii*]	6	1	1	185	20.9
AEP19907.1	ribosomal protein L14 [*Exiguobacterium chiriqhucha*]	19	4	1	122	13.3
CDK35693.1	Translation initiation factor IF-3 [*Lactobacillus salivarius* cp400]	7	2	1	189	21.6
HBU67662.1	50S ribosomal protein L4 [*Lactobacillus* sp.]	6	1	1	207	22.4
ASC08568.1	Elongation factor Ts [*Pediococcus pentosaceus*]	4	2	2	292	31.9
QHO68129.1	Foldase protein PrsA [*Pediococcus pentosaceus*]	3	3	1	295	32.5
ANS61171.1	penicillin-binding protein [*Streptococcus thermophilus*]	1	4	1	787	86.4

GBD71813.1	phosphoenolpyruvate--protein phosphotransferase [*Tetragenococcus halophilus subsp. halophilus*]	2	1	1	575	63.6
AEJ43046.1	6-phosphogluconate dehydrogenase, decarboxylating [*Alicyclobacillus acidocaldarius subsp. acidocaldarius* Tc-4-1]	4	3	1	480	53.2
ASV96427.1	molecular chaperone DnaK [*Enterococcus durans*]	4	2	1	610	65.7
ADM08624.1	glyceraldehyde-3-phosphate dehydrogenase [*Parvularcula bermudensis* HTCC2503]	6	2	1	335	35.9
ARW19542.1	Elongation factor Tu [*Pediococcus pentosaceus*]	13	16	1	395	43.3
GBD70157.1	60 kDa chaperonin [*Tetragenococcus halophilus subsp. halophilus*]	5	2	2	546	57.7
PAY44296.1	chain-length determining protein [*Lactobacillus salivarius*]	5	3	1	265	29.2
AYC11341.1	Penicillin-binding protein 1F [*Lactobacillus salivarius*]	3	5	2	714	79.3
